# Supplementary material for: One-quarter of freshwater fauna threatened with extinction
Source: Nature. 2025 Jan 8;638(8049):138–45. doi: 10.1038/s41586-024-08375-z (PMC11798842; doi:10.1038/s41586-024-08375-z)
Supplement: Supplementary file 1 — This file contains Supplementary Note 1 and Supplementary Table 1 [file 41586_2024_8375_MOESM1_ESM.pdf]

nature portfolio

https://doi.org/10.1038/s41586-024-08375-z

Supplementary information

One-quarter of freshwater fauna threatened with extinction

In the format provided by the authors and unedited

Title: One quarter of freshwater fauna threatened with extinction

Authors: Sayer et al.

SUPPLEMENTARY INFORMATION

Supplementary Note 1

a) List of assessors – freshwater fishes

The following individuals were assessors for at least one freshwater fish Red List assessment used in this study:

Abdulqader, E., Abraham, R., Acero, A., Acosta, A.K.M., Adams, A., Adeofe, T.A., Aguilera Socorro, O., Ahmad, A.B., Ahmad, I., Aiken, K.A., Akhilesh, K.V., Akinsola, O., Akinyi, E., Alam, S., Albieri, R.J., Alcantara, A.J. , Alcantara, M., Al-Husaini, M., Ali, A., Al-Khalaf, K., Allan, H., Allen, D.J., Allen, G.R., Allibone, R., Almada-Villela, P., Almukhtar, M., Alnazry, H., Alonso, F., Alonso, J.C., Alvarado, J.J., Amaoka, K., Ame, E.C., Andayog, A.S., Anderson, E., Anderson, W., Angeles, I.J.P., Angulo, A., Araujo Q., A., Arceo, H., Ardon, D., Arguello, P., Armbruster, J.W., Arroyave, J., Arthington, A., Arunachalam, M., Awaïss, A., Aylesworth, L., Azeroual, A., Bactong Jr., M.A., Baigun, C., Baigún, C., Baird, I., Baker, C., Ballad, E.L., Ballen, G.A., Barbhuiya, A.H., Barkhuizen, L.M, Barraza, E., Barrientos, C., Bartnick, S., Bayona, J.D.R., Bearez, B., Béarez, P., Beatty, S., Becker, L., Belga, P.B., Bello P., J.A., Bessonart, J., Betancur, R., Bezerra, I., Bianco, P.G., Bice, C., Bigirimana, C., Bills, R., Bin Ali, A., Birge, T.L., Bishop, J., Blanco-Parra, M.P., Boden, G., Bogutskaya, N., Bohlen, J., Bolden, S.K., Bonfil-Sanders, R., Borsa, P., Boseto, D., Bouchereau, J.-L., Bousso, T., Bragg, C., Brancolini, F., Brejão, G.L., Brenner, J., Brick Peres, M., Briones Bell-lloch, A., Britto, M.R., Britz, R., Bronzi, P., Brooks, E., Brooks, S., Brown, C., Brown, J., Bru, E., Bruce, A., Bruch, R., Buccat, F.G.A., Buddo, D., Bussing, W., Butler, G., Buxton, C.D., Caires, R.A., Calegari, B.B., Camara, K.D., Camara, Y.H., Cambray, J., Cameron, C., Cao, L., Capuli, E.,

Cardenosa, D., Cardinale, M., Cardozo, V., Carlson, J., Carmona, J., Carpenter, K.E., Carrasco, J.C., Caruso, J., Carvajal, F., Carvajal-Vallejos, F.M., Carvalho, F.R., Castellanos-Morales, C.C.M., Castro, M.G., Cecilio, M.A.F., Celestin, B., Chakona, A., Chakrabarty, P., Champion, P., Chang, S.-K., Chao, L., Charles, R., Chartrain, E., Charvet, P., Charvet-Almeida, P., Chaudhry, S., Chavez Mendoza, G., Chebanov, M., Chen, X., Chen, X.-Y., Cheok, J., Chevis, M., Chiaramonte, G., Chocano, L., Chua, K.W.J., Chuctaya, J., Chura-Cruz, R., Cissoko, K., Claro, R., Clements, K.D., Closs, G., Clunie, P., Cockayne, B., Coetzer, W., Cole, K., Coleman, R., Colin, N., Collen, B., Collette, B.B., Congiu, L. , Contreras MacBeath, T., Contreras-Balderas, S., Conway, K.W., Cook, R., Cooke, A., Cooke, R., Cooper, D., Correa, E., Correa, V., Cortijo Villaverde, A.M., Cotto, A., Cotton, A., Cowan, J., Craig, M.T., Crichigno, S., Crivelli, A.J., Crook, V., Crossman, J., Crow, S., Cui, K., Cussac, V., da Costa, L., da Silva, F.M.S., Dahanukar, N., Daniels, A., Dankwa, H., Darwall, W.R.T., David, B., David, E.B., de Almeida, M.P., de Alwis Goonatilake, S., De Bruyne, G., de la Maza-Benignos, M., de Morais, L., de Zeeuw, M.P., Decru, E., Deligero, R., Derrick, D., Devi, R., Dey, S.C., Dharmadi, Di Dario, F., Di Natale, A., Diallo, I., Díaz, G.Q.E., Dicken, M., Diedericks, G.J., Diop, M., Diouf, K., Djiman, R., Doherty, P., Domínguez, O., Dominici-Arosemena, A., DoNascimiento, C.D., Dooley, J., dos Santos, S.A., Douglas, R., Doyola, M.C., Drioli, M., Du, L.-N., Duchi, A., Dulvy, N.K., Ebert, D.A., Ebner, B., Echevarría, G., Edgar, G., Elias, D., Ellender, B.R., Ellis, I.M., Elvira, B., Engelbrecht, J., Entsua-Mensah, M., Erk'akan, F., Eschmeyer, W., Espino Ciudad, J., Espinosa Pérez, H., Espinosa, H., Espinoza, E., Espinoza, M., Espinoza, T., Evans, S.W., Everett, B., Eza, N.D., Fadré, N.N., Fahmi, Fan, L., Faria, V., Faustino-Fuster, D.R., Fazli, H., Feary, D., Fernado, M., Fernandes, P., Fernandez-Auderset, J., Fernando, E., Ferretti, F., Findley, L., Florin, A.-B., Fontenelle, J.P., Ford, M., Fordham, S., Fox, D., Fox, W., Franklin, P., Fraser, T., Frederico, R.G., Fredou, F.L., Freeman, R., Freyhof, J., Fricke, R., Friedrich, T., Fritzsche, R., Fuchs, D.V., Fuentes, C., Fujii, I., Furtado, M., Gaerlan, R.S.P.,

Gante, H., Gapuz, A.V., García Dávila, C., García Vásquez, A., Garcia, M.P.C., García-Machado, E., Garrone Neto, D., Gatlabayan, L.V., Gaughan, D., Gautama, D.A., Geelhand, D., Gessner, J., Getahun, A., Giarrizzo, T., Gilligan, D., Gilmore, G., Gimena, R.V., Gimenez Dixon, M., Gobo, E., Góes de Araújo, M.L., Gollock, M., Gómez Balandra, A., Gon, O., Gonzales, J.C., Gonzalez, J.C.T., González, R., Goren, M., Górski, K., Gotch, T., Gouws, G., Grant, I., Grant, M.I., Greenfield, D., Grijalba Bendeck, L., Grubbs, D., Guerrero, R.D. III, Guindon, K., Guino-o, R.S. II, Guzman-Mora, A.G., Hadiaty, R.K., Hammer, M., Hammerson, G.A., Hanssens, M., Haque, A.B., Hardie, S., Hardy, G., Harrison, A., Harrison, I.J., Harry, A.V., Hartmann, S., Hasan, M.E., Hasan, V., Hasan, Z., Hasegawa, K., Hastings, P., Hata, H., Haxton, T., Hendrickson, D., Herler, J., Herman, K., Herrera-Collazos, E.E., Hew, S., Hidalgo del Aguila, M., Hildebrand, L., Hilton, E., Hitchmough, R., Ho, H., Ho, J.K.I., Hoese, D., Hoffman, A., Hogan, Z., Horodysky, A., Hosoya, K., Hsu, H., Huckstorf, V., Hughes, A., Impson, D., Imran, M., Inui, R., Iwamoto, T., Iwatsuki, Y., Jaafar, Z., Jabado, R.W., Jacoby, D., Jan, A., Janekikarn, S., Jelks, H., Jenkins, A., Jensen, O., Jha, B.R., Jiang, W.-S., Jiang, Y.-E., Jiang, Z.-G., Jimenez-Prado, P., Jimenez-Segura, L., Jing, L., Johnson, J.A., Jones, G.C.A., Jordaan, M., Jordan, G., Juan Jorda, M., Juffe Bignoli, D., Kacoliris, F., Kadarusman, Kadye, W.T., Kaifu, K., Kakkasery, F., Kanao, S., Kanyerere, G.Z., Karatash, A., Karimov, B., Katwate, U., Kaufman, L., Kaunda, E., Kaymaram, F., Kazembe, J., Keith Diagne, L., Keith, P., Kelez, S., Kennard, M., Kerezsy, A., Kern, P., Kesner-Reyes, K., Kishe, M., Kishore, R., Knapp, L.W., Knudsen, S., Ko, M.-H., Koeck, M., Koehn, J., Kolkolo, U., Konings, A., Kosgei, G., Koster, W., Kotagama, O., Kottelat, M., Krajangdara, T., Kubheka, S., Kuhajda, B., Kullander, F., Kullander, S.O., Kyne, P.M., Lalèyè, P.A., Lambarri Martínez, C., Lambeth, S., Lanzuela, N., Lardizabal, C.C., Larson, H., Lasso, C., Lasso-Alcalá, O.M., Lawrence, A., Le Feuvre, M.C., Lea, B., Lea, R.N., Leander, N.J.S., Lear, K.O., Lee, S.-H., Leis, J.L., Leurs, G.H.L., Lian, G., Liao, L., Lieschke, J., Lim, K., Lima, F., Lindeman, K., Ling, N., Lintermans,

M., Linus, E.N., Liotta, J., Liu, M., Loeb, M., Loh, K.-H., Loiselle, P.V., Lopez, G., Loureiro, M., Low, B.W., Lubbe, A., Lumbantobing, D., Lyons, T.J., Maake, P., MacDonald, T., Madhusoodana Kurup, B., Magombo, Z.L.K., Mailautoka, K., Mailosi, A., Maiz-Tome, L., Makocho, P., Maldonado, M., Maldonado-Ocampo, J., Mallinson, J., Mamalangkap, M.D., Mamilov, N., Manimekalan, A., Mann, B.Q., Manosalva, A., Marcante, F., Marceniuk, A.P., Marechal, J.-P., Marr, S., Marrero, C., Marshall, B.E., Martin, C.H., Martin, K., Martínez, E.O., Martins, J., Matamoros, W.A., Mateos, M., Mathwin, R., Matsuura, K., Mazungula, N., Mbye, E.M., McBride, R., McCosker, J., McEachran, J.D., McMahan, C., Mejía Guerrero, O., Mendonca, E., Mendonça, J.T., Mercado Silva, N., Mesa-Salazar, L., Metcalfe, K., Mikolji, I., Minotti, P., Miyazaki, Y., Mizrahi, M., Moelants, T., Mohanraj, G., Mohd Arshaad, W., Mojica, J.I., Mokodongan, D.F., Møller, P.R., Moncada Gallardo, T., Montiero, V., Moore, G., Moore, M., Morales-Saldaña, J.M., Moretzsohn, F., Morgan, D.L., Morgan, S.K., Moser, M., Motomura, H., Moy, K., Moya Vásquez , L., Muallil, R., Mugue, N., Mukai, T., Mungkaje, A., Muñoz-Ramírez, C., Munroe, T.A., Murdy, E., Musinguzi, L., Musschoot, T., Mutia, M.T.M., Muyot, M.C., Myers, R., Nacua, S.S., Nagy, B., Nair, R., Nakajima, J., Nanola, C., Naranjo-Elizondo, B., Narejo, N., Natakimazi, G., Natugonza, V., Nedreaas, K., Nelson, J., Nelson, R., Nelson-Stastny, W., Ng, H.H., Ngobela, T., Nguku, J.K., Nguyen Van, Q. , Nguyen, T.H.T., Nielsen, J., Norris, S., Ntakimazi, G., Nunes, J., Nunoo, F., Nyagweth, W., Nyingi, D., Nzeyimana, L., O'Brien, G., Odhiambo, E.A., Olaosebikan, B.D., Ornelas García, P., Ortega Torres, H., Ouyang, L., Oxenford, H., , Pacoureau, N., Padovani-Ferreira, B., Pal, M., Palla, H.P., Palmer-Newton, A., Panduro, M., Parenti, L., Parido, L. , Paxton, B.R., Pearce, L., Perea Sicchar, C., Perez, A., Pérez-Miranda, F., Pethiyagoda, R., Petrtýl, M., Pezold, F., Phelps, Q., Phillips, N.M., Phiri, T.B., Pike, C., Pina Amargos, F., Pinder, A., Pinto de Almeida, M., Polanco Fernandez, A., Pollard, D.A., Pollom, R., Ponce de León, J., Pourkazemi, M., Povedano, H., Provenzano-Rizzi, F., Qadir, A., Qiwei, W., Quartey, R., Quiazon, K.M.A.,

Quilang, J.P., Quintana, Y., Quintero-T., E., Quiroga, S., Qureshi, I., Raadik, T., Rachinski, T., Raffan, O., Rafique, M., Raghavan, R., Rainboth, W., Ram, M., Ramzan, M., Rand, P.S., Ravelomanana, T., Rayamajhi, A., Reategui Ocampo, D., Rebancos, C.M., Reis, R., Rema Devi, K.R., Reyes Ramírez, C., Reyes, A.T., Rider, S., Rigby, C.L., Rincón, G., Rivas, M., Roberts, D., Robertson, R., Rocha, L.A., Rochard, E., Rodrigues, A.M.T., Rodrigues, J., Rodriguez, C., Rodríguez-Machado, S., Rodríguez-Olarte, D., Rodríguez-Silva, R., Rojas, P., Rosa, R.S., Roux, F., Ruban, G., Russell, B., Saddlier, S., Sagna, A., Salarpouri, A., Salas, E., Salvador, G.N., Sammons, S., Sampson, C., Sánchez Riveiro, H., Sanchez-Duarte, P., Santana, F.M., Santos, M., Santos, S., Sarmiento, J., Sasaki, K., Sayer, C., Schlupp, I., Schmarr, D., Schmidt, R., Schmitter-Soto, J., Schneider, E.V.C., Schumann, M., Scott, J.A., Scott, K., Seah, Y.G., Sedberry, G., Seidu, I., Seyha, L., Shah, N.H.A., Shaji, C.P., Shao, K.-T., Shao, W., Sharley, J., Shechonge, A., Shen, J.-Z., Shenker, J., Shibukawa, K., Shlyakhov, V., Sianipar, A., Sidharthan, A., Sidibé, A., Sidlauskas, B.L., Sifundza, D., Simeon, B., Simons, J., Simpfendorfer, C., Simpson, N.J., Singh, L.K., Singh-Renton, S., Skelton, P.H., Slobodian, V., Smith, D.G., Smith, K., Smith-Vaniz, B., Smith-Vaniz, W.F., Snoeks, J., Soliman, V.S., Song, Z., Sorensen, M., Soto Galera, E., Soto, J.M.R., Sparks, J.S., Sternberg, D., Stewart, D.J., Stoessel, D., Storey, A.W., Stoumboudi, M., Suharti, S., Superina, M., Surrey, G. , Swartz, E.R., Sylla, M., Takaku, K., Tambihasan, A.M., Tamo, A., Tan, H.H., Taniguchi, Y., Taphorn, D.C., Tencatt, L.F.C., Tenzin, K., Than Tun, M., The Anh, B., Thinh, D.V., Tighe, K., Tognelli, M.F., Tolan, J., Tomelleri, J.R., Tonkin, Z., Torgersen, K., Tornabene, L., Torres, A.G., Torres, F. , Torres, Y.T.P., Torres-Pineda, P., Tous, P., Turan, C., Turner, G., Tweddle, D., Twongo, T.K., Tyler, J., Ulmo-Díaz, G., Unmack, P., Uozumi, Y., Usma, S., Vaidyanathan, T., Valderrama, M., Valdes Gonzales, A., Valenti, S.V., Valenzuela Mendoza, L., Valverde, D., Vamosi, S., Van Damme, P., van der Heiden, L.B., Van Der Meulen, D., Van der Walt, R., van Heiden, A., Van Tassell, J.L., Van Veen, F., Van, N.S., VanderWright, W.J., Varella, H.R.,

Veale, L. , Vega-Cendejas, M., Velasquez Quispe, M.A., Velasquez, M., Vera-Alcaraz, H.S., Vidthayanon, C., Vieira, J.P., Villa-Navarro, F.A., Villanueva, J.A., Villanueva, T.R., Villarao, M.C., Villwock de Miranda, L., Vishwanath, W., Vo, V.Q., Volta, P., von der Heyden, S., Vreven, E., Wager, R., Wakhida, Y., Walsh, C., Wang, X., Ward, R., Watanabe, K., Watters, B., Webb, M., Wedderburn, S.D., Weerts, S.P., West, D., Weyl, O., Whiterod, N., Williams, A.B., Williams, J.T., Williot, P., Wiswedel, S., Witte, F., Won, S.-Y., Wong, L., Woodford, D., Wueringer, B.E., Yang, B., Yang, J., Yang, J.-X., Yeeting, B., Yépez, V., Yuan, L-Y., Yum, Y.-W., Yuneni, R.R., Zafarullah, M., Zampatti, B., Zhang, E., Zhang, J., Zhao, H.H., Zhao, Y., Zhou, W., Zhuang, H., and Zhuang, X.

The following organisations were assessors for at least one freshwater fish Red List assessment used in this study:

FishBase Royal Museum for Central Africa, Instituto Chico Mendes de Conservação da Biodiversidade (ICMBio), NatureServe, and World Conservation Monitoring Centre.

b) List of assessors – odonates

The following individuals were assessors for at least one odonate Red List assessment used in this study:

Abbott, J.C., Amaya-Vallejo, V., Babu, R., Beatty, C.D., Bedjanič, M., Bernard, R., Bilane, D.B., Bota-Sierra, C.A., Boudot, J.-P., Cano-Cobos, Y., Cashatt, E., Chung, H.-Y., Clausnitzer, V., Cordero Rivera, A., Díaz-Martínez, C., De Marmels, J., Dijkstra, K.-D.B., Do, C.D., Donnelly, N., Donnelly, T., Dow, R.A., Ferreira, S., Florez, C., Futahashi, R., Günther, A., González, E.M., González-Soriano, E., Guillermo-Ferreira, R., Guzmán Ojeda, R.J., Hämäläinen, M., Haber, W.A., Harding, K.M., Hawking, J., Hoffmann, J., Hong, M.-H., Ikemeyer, D., Inoue, K., Jović, M., Juen, L., Kakkasery, F., Kalkman, V.J., Kamaludin, N., Karube, H., Kipping, J., Kompier, T., Kosterin, O.E., Lee, Y.-S., Lim, C.-S., Lorenzo-Carballa,

M.O., Lozano, F., Malikova, E.M., Manh, C.D., Marinov, M., Martens, A., Mauffray, W., Minot, M., Mitra, A., Montes-Fontalvo, J.M., Moore, S., Muzón, J., Nguyen, T.H.T., Nishihara, S., Novelo-Gutierrez, R., Ogbogu, S., Ortega-Salas, H., Pérez-Gutiérrez , L.A., Pal, M., Palacino, F., Park, S.-H., Paulson, D.R., Perez, L., Phan, Q., Pinto, Ã,.P., Polhemus, D.A., Pryce, D., Rache, L., Rachman, H.T., Realpe, E., Reels, G., Richards, S., Rowe, R., Saade, E., Sahlén, G., Samraoui, B., Samways, M.J., Sandoval-H, J., Sasamoto, A., Schütte, K., Schneider, T., Schneider, W., Sharma, D., Sharma, G., Simaika, J., Subramanian, K.A., Suhling, F., Sumanapala, A.P., Tchibozo, S., Tennessen, K., Theischinger, G., Tong, X., Torres Cambas, Y., Torres-Pachon, M., Vilela, D., Villanueva, R.J.T., Vivas Santeliz, J.J., von Ellenrieder, N., Wasscher, M., Weigel Muñoz, S., Wildermuth, H., Wilson, K.D.P., and Zhang, H.

c) List of assessors – freshwater decapod crustaceans

The following individuals were assessors for at least one freshwater decapod crustacean Red List assessment used in this study:

Adams, S., Alvarez, F., Anker, A., Austin, C.M., Ayhong, S., Bahir, M.M., Bergey, E., Buckup, L., Buhay, J., Bunn, J., Burnham, Q., Cai, X., Cai, Y., Cordeiro, J., Coughran, J., Crandall, K.A., Cumberlidge, N., Daniels, S., Dawkins, K.L., De Grave, S., DiStefano, R., Doran, N., Edsman, L., Elliott, R., Esser, L.J., Eversole, A.G., Füreder, L., Furse, J., Gherardi, F., Hamr, P., Holdich, D., Horwitz, P., Iliffe, T.M., Johnson, D., Johnston, K., Jones, C., Jones, J.P.G., Jones, R.L., Jones, T., Kawai, T., Klotz, W., Lawler, S., López-Mejía, M., Machino, Y., Mantelatto, F., Marijnissen, S.A.E., Mathews, L.M., McCormack, R.B., Moler, P., Naruse, T., Ng Kee Lin, P., Page, T., Parkyn, S., Pedraza Lara, C., Peer, N., Pethiyagoda, R., Randrianasolo, H., Rasamy, J., Reynolds, J., Richardson, A., Rogers, C., Schubart, C., Schultz, M., Schuster, G.A., Shy, J., Sibley, P., Skelton, C., Souty-Grosset, C., Taylor, C.A., Thoma,

R.F., Van Der Colff, D., Villalobos, J., von Rintelen, K., Walls, J., Walsh, T., Wingfield, M., Wowor, D., and Yeo, D.

Supplementary Table 1

Regional sub-projects undertaken as part of the global assessment efforts. The three main tasks in these projects (i) Red List assessor training; ii) data compilation and Red Listing assessment drafting; iii) and Red List assessment review occurred either **remotely** (i.e., species experts working online and individually on a task), during **virtual workshops** (i.e., a group of species experts and facilitator(s) meeting online to complete a task), and/or during regional **in-person workshops** (i.e., a group of species experts and facilitator(s) meeting in-person, normally within the region of interest, to complete a task).

a) Freshwater fishes

Start Year	End Year	Region	Red List assessor Training	Data compilation and Red List assessment drafting	Red List assessment review
2003	2004	East Africa	In-person workshop	Remote	In-person workshop
2004	2005	Mediterranean	In-person workshop	Remote	In-person workshop
2005	2005	Mongolia	Remote	Remote	In-person workshop
2005	2006	Southern Africa	In-person workshop	Remote	In-person workshop
2005	2006	Western Africa	In-person workshop	Remote	In-person workshop
2006	2007	North Africa	In-person workshop	Remote	In-person workshop
2007	2007	Europe	Remote	Remote	In-person workshop
2009	2010	Eastern Himalayas	In-person workshop	Remote	In-person workshop
2009	2010	North-East Africa	Remote	Remote	Remote
2009	2011	Indo-Burma	In-person workshop	Remote	In-person workshop
2010	2011	Central Africa	In-person workshop	Remote	In-person workshop
2010	2011	Western Ghats	In-person workshop	Remote	In-person workshop
2011	2012	New Zealand	In-person workshop	In-person workshop	In-person workshop
2011	2012	Oceania	In-person workshop	Remote	In-person workshop
2012	2013	North America	Remote	Remote	Remote
2012	2015	Arabian Peninsula	Remote	Remote	In-person workshop; Remote

2013	2014	Eastern Mediterranean	Remote	Remote	In-person workshop
2014	2015	Tropical Andes	Remote	Remote	In-person workshop
2015	2016	Lake Victoria Basin	Remote	Remote	In-person workshop
2016	2017	Madagascar	Remote	Remote	In-person workshop
2016	2019	Malili Lakes	In-person workshop	Remote	Remote
2017	2019	Japan	In-person workshop	In-person workshop	In-person workshop
2018	2018	Lake Malawi/Nyasa/Nyassa Basin	Remote	Remote	In-person workshop
2018	2019	Australia	Remote	In-person workshop	In-person workshop
2018	2019	Mexico	Remote	Remote	In-person workshop
2018	2021	Sunda Basin	In-person workshop	Remote	In-person workshop; Remote
2018	2023	Europe	In-person workshop	In-person workshop; Remote	In-person workshop; Remote
2018	2023	Brazil	Remote	Remote (in collaboration with the National Red List of Brazil)	Remote
2019	2019	Sri Lanka	Virtual workshop	Remote	In-person workshop
2019	2020	Pakistan	Remote	Remote	In-person workshop; Remote
2019	2020	Russia and Central Asia	Remote	Remote	In-person workshop; Remote
2019	2020	Western Africa	Remote	Remote	Remote
2019	2021	New Guinea	Remote	Remote	In-person workshop; Remote
2020	2022	Philippines	Remote	Remote	Remote
2020	2023	Korean Peninsula	In-person workshop	Remote (in collaboration with National Red List of Republic of Korea)	In-person workshop (in collaboration with National Red List of Republic of Korea); Remote
2020	2023	South America (excluding Brazil)	Remote	Remote	Remote
2020	2023	South American freshwater stingrays	Virtual workshop	Virtual workshop	Remote
2020	2023	India	Remote	Remote	Remote
2020	2023	China	Remote	Remote	Virtual workshop
2021	2021	Caribbean	Remote	Virtual workshop; Remote	Remote
2021	2022	Uganda	Remote	Remote	Remote

b) Odonates

Start Year	End Year	Region	Red List assessor Training	Data compilation and Red List assessment drafting	Red List assessment review
2003	2004	East Africa	In-person workshop	Remote	In-person workshop
2004	2005	Mediterranean	In-person workshop	Remote	In-person workshop
2005	2006	Southern Africa	In-person workshop	Remote	In-person workshop
2005	2006	Western Africa	In-person workshop	Remote	In-person workshop
2006	2007	North Africa	In-person workshop	Remote	In-person workshop
2009	2010	Europe	Remote	Remote	In-person workshop
2009	2010	Eastern Himalayas	In-person workshop	Remote	In-person workshop
2009	2010	North-East Africa	Remote	Remote	Remote
2009	2011	Indo-Burma	In-person workshop	Remote	In-person workshop
2010	2011	Central Africa	In-person workshop	Remote	In-person workshop
2010	2011	Western Ghats	In-person workshop	Remote	In-person workshop
2011	2012	New Zealand	In-person workshop	In-person workshop	In-person workshop
2011	2012	Oceania	In-person workshop	Remote	In-person workshop
2012	2015	Arabian Peninsula	Remote	Remote	In-person workshop
2013	2014	Eastern Mediterranean	Remote	Remote	In-person workshop
2014	2014	Italy	In-person workshop	In-person workshop	In-person workshop
2014	2015	Tropical Andes	Remote	Remote	In-person workshop
2015	2016	Lake Victoria Basin	Remote	Remote	In-person workshop
2015	2016	France	In-person workshop	In-person workshop	In-person workshop
2016	2016	Brazil	In-person workshop	Remote	Remote
2016	2017	Madagascar	Remote	Remote	In-person workshop
2016	2019	Malili Lakes	In-person workshop	Remote	Remote

2017	2017	Colombia	In-person workshop	Remote	Remote
2017	2019	Japan	In-person workshop	In-person workshop	In-person workshop
2018	2018	Lake Malawi/Nyasa/Nyassa Basin	Remote	Remote	In-person workshop
2021	2023	Europe	Virtual workshop	Virtual workshop; Remote	Virtual workshop; Remote
