# Supplementary material for: One-quarter of freshwater fauna threatened with extinction
Source: Nature. 2025 Jan 8;638(8049):138–45. doi: 10.1038/s41586-024-08375-z (PMC11798842; doi:10.1038/s41586-024-08375-z)
Supplement: Supplementary file 2 — Reporting Summary [file 41586_2024_8375_MOESM2_ESM.pdf]

nature portfolio

Corresponding author(s): Catherine A. Sayer

Last updated by author(s): Sep 23, 2024

Reporting Summary

Nature Portfolio wishes to improve the reproducibility of the work that we publish. This form provides structure for consistency and transparency in reporting. For further information on Nature Portfolio policies, see our Editorial Policies and the Editorial Policy Checklist.

Statistics

For all statistical analyses, confirm that the following items are present in the figure legend, table legend, main text, or Methods section.

n/a	Confirmed	
☐	☒	The exact sample size (*n*) for each experimental group/condition, given as a discrete number and unit of measurement
☒	☐	A statement on whether measurements were taken from distinct samples or whether the same sample was measured repeatedly
☐	☒	The statistical test(s) used AND whether they are one- or two-sided<br>*Only common tests should be described solely by name; describe more complex techniques in the Methods section.*
☒	☐	A description of all covariates tested
☒	☐	A description of any assumptions or corrections, such as tests of normality and adjustment for multiple comparisons
☐	☒	A full description of the statistical parameters including central tendency (e.g. means) or other basic estimates (e.g. regression coefficient) AND variation (e.g. standard deviation) or associated estimates of uncertainty (e.g. confidence intervals)
☐	☒	For null hypothesis testing, the test statistic (e.g. F, t, r) with confidence intervals, effect sizes, degrees of freedom and P value noted<br>*Give P values as exact values whenever suitable.*
☒	☐	For Bayesian analysis, information on the choice of priors and Markov chain Monte Carlo settings
☒	☐	For hierarchical and complex designs, identification of the appropriate level for tests and full reporting of outcomes
☒	☐	Estimates of effect sizes (e.g. Cohen's *d*, Pearson's *r*), indicating how they were calculated

Our web collection on statistics for biologists contains articles on many of the points above.

Software and code

Policy information about availability of computer code

Data collection	No code was used for the data collection in this study.
Data analysis	We used the following open source software to perform the surrogacy analyses: R (v4.3), Zonation (v4, which includes the CAZ and ABF algorithms) and zonator (v0.6.0). Custom R scripts were developed to work with the software Zonation and are available at: https://zenodo.org/doi/10.5281/zenodo.10286099.<br>We also used Microsoft Excel to analyse tabular data on extinction risk, habitats, and threats. Chi-squared tests were also performed in Microsoft Excel (Version 2408).

For manuscripts utilizing custom algorithms or software that are central to the research but not yet described in published literature, software must be made available to editors and reviewers. We strongly encourage code deposition in a community repository (e.g. GitHub). See the Nature Portfolio guidelines for submitting code & software for further information.

Data

Policy information about availability of data

All manuscripts must include a data availability statement. This statement should provide the following information, where applicable:

- Accession codes, unique identifiers, or web links for publicly available datasets
- A description of any restrictions on data availability
- For clinical datasets or third party data, please ensure that the statement adheres to our policy

Taxonomic data for freshwater fishes are from Eschmeyer's Catalog of Fishes (http://researcharchive.calacademy.org/research/ichthyology/catalog/fishcatmain.asp) and for odonates are from World Odonata List (https://www.pugetsound.edu/slater-museum-natural-history-0/biodiversity-resources/insects/dragonflies/world-odonata-list).
All IUCN Red List assessment data are publicly available on The IUCN Red List of Threatened Species website (www.iucnredlist.org). Occasionally, where a species may be under threat from over-collection, sensitive spatial data are not publicly available. All tabular and spatial data from the IUCN Red List will be summarised and made available at www.iucnredlist.org/resources/data-repository prior to publication of the manuscript.
Baseline water stress data ('Aqueduct Water Stress Projections Data') are available from the Aqueduct Water Risk Atlas here: https://www.wri.org/data/aqueduct-water-stress-projections-data. Baseline nitrogen data ('Global - Nitrate-nitrite in Surface Water') are available from the World Bank catalogue here: https://datacatalog.worldbank.org/search/dataset/0038385/Global---Nitrate-nitrite-in-Surface-Water.

Research involving human participants, their data, or biological material

Policy information about studies with human participants or human data. See also policy information about sex, gender (identity/presentation), and sexual orientation and race, ethnicity and racism.

Reporting on sex and gender	This research does not involve human participants, their data, or biological material.
Reporting on race, ethnicity, or other socially relevant groupings	This research does not involve human participants, their data, or biological material.
Population characteristics	This research does not involve human participants, their data, or biological material.
Recruitment	This research does not involve human participants, their data, or biological material.
Ethics oversight	This research does not involve human participants, their data, or biological material.

Note that full information on the approval of the study protocol must also be provided in the manuscript.

Field-specific reporting

Please select the one below that is the best fit for your research. If you are not sure, read the appropriate sections before making your selection.

☐ Life sciences ☐ Behavioural & social sciences ☒ Ecological, evolutionary & environmental sciences

For a reference copy of the document with all sections, see nature.com/documents/nr-reporting-summary-flat.pdf

Ecological, evolutionary & environmental sciences study design

All studies must disclose on these points even when the disclosure is negative.

Study description	This study examines the extinction risk, distribution, key habitats, and drivers of threat of freshwater species (decapod crustaceans, fishes, and odonates) globally, in comparison to the status of tetrapods (amphibians, birds, mammals, and reptiles) based on data from The IUCN Red List of Threatened Species. Additionally, the study includes an analysis of the effectiveness of threatened tetrapods and an abiotic factor (water stress) as surrogates for the conservation of threatened freshwater species.
Research sample	The research sample was assessments of species for The IUCN Red List of Threatened Species (www.iucnredlist.org). For each species, these assessments include information on taxonomy, distribution (include a range map), population, habitats and ecology, use and trade, threats, conservation and research actions, and a Red List category of extinction risk (assigned based on Red List criteria). Data were from the 2022-2 version of the IUCN Red List, downloaded in March 2023, with additional unpublished data for a subset of freshwater fishes.<br><br>Additional datasets used were Eschmeyer's Catalog of Fishes (http://researcharchive.calacademy.org/research/ichthyology/catalog/fishcatmain.asp) and World Odonata List (https://www.pugetsound.edu/slater-museum-natural-history-0/biodiversity-resources/insects/dragonflies/world-odonata-list).<br><br>See the Methods for further details.
Sampling strategy	We aimed to include data on all formally described species of freshwater decapod crustaceans, fishes, and odonates. However,

Sampling strategy	primarily due to new descriptions since regional assessment efforts took place, the study included data on over 80% of all described freshwater species combined in the taxonomic groups of interest. This value is considered "comprehensively assessed" on the IUCN Red List and sufficient to represent the status of a taxonomic group. See the Methods for further details.
Data collection	Data were collected in the IUCN Species Information Service database (https://sis.iucnsis.org/apps/org.iucn.sis.server/SIS/index.html). Initial data compilation was primarily by species experts working remotely. This was then followed by workshops (primarily in person) where groups of species experts and facilitators with expertise in the IUCN Red List Categories and Criteria met to review and approve the data. Assessors and reviewers are listed in the credits of each assessment. The final assessments were then re-reviewed and approved by staff in the IUCN Red List Unit. See the Methods and Supplementary Information for further details.
Timing and spatial scale	Data collection took place between 2003 and 2023 through a series of regional assessment efforts. The frequency and duration of each assessment effort was dependent on funding available. Full details of timelines associated with each regional assessment effort can be found in the Supplementary Information. The data are global in scale, covering all areas where freshwater decapod crustaceans, fishes, and odonates are known to occur.
Data exclusions	No data were intentionally excluded from the analysis. However, some species were not included if, for example, they were formally described after their native region was assessed.
Reproducibility	Each assessment underwent two reviews. First, at least one independent scientist familiar with each species reviewed the assessment to ensure the data presented were correct and complete, and that the Red List Criteria had been applied appropriately. Once each assessment had passed this first stage of the review, including revision (if necessary), staff from the IUCN Red List Unit reviewed the assessments to ensure that the Red List Criteria had been applied appropriately, and the documentation standards had been met. Once each assessment had passed this second stage of the review, again including revision (if necessary), they were considered finalised and set for publication on the IUCN Red List website. A subset of the freshwater fish species used in this analysis had undergone only the first step of the review process described above at the time of writing. No attempts have been made to reproduce the original assessments.
Randomization	No randomised groups were used in the study as we aimed to cover all formally described freshwater decapod crustaceans, fishes, and odonates at the time of assessment. There were no relevant covariates to consider in the analysis.
Blinding	Blinding was not relevant to this study, which did not involve experimental analysis.

Did the study involve field work? ☐ Yes ☒ No

Reporting for specific materials, systems and methods

We require information from authors about some types of materials, experimental systems and methods used in many studies. Here, indicate whether each material, system or method listed is relevant to your study. If you are not sure if a list item applies to your research, read the appropriate section before selecting a response.

Materials & experimental systems

n/a	Involved in the study
☒	☐ Antibodies
☒	☐ Eukaryotic cell lines
☒	☐ Palaeontology and archaeology
☒	☐ Animals and other organisms
☒	☐ Clinical data
☒	☐ Dual use research of concern
☒	☐ Plants

Methods

n/a	Involved in the study
☒	☐ ChIP-seq
☒	☐ Flow cytometry
☒	☐ MRI-based neuroimaging

Plants

Seed stocks	This research does not involve plants.
Novel plant genotypes	This research does not involve plants.
Authentication	This research does not involve plants.
